# *YWHAZ* and *TBP* are potential reference gene candidates for qPCR analysis of response to radiation therapy in colorectal cancer

**DOI:** 10.1038/s41598-023-39488-6

**Published:** 2023-08-09

**Authors:** Shin Kim, Jee Young Park, Hye Won Lee, Sung Uk Bae, Kyeong Eui Kim, Sang Jun Byun, Incheol Seo

**Affiliations:** 1 Department of Immunology, Keimyung University School of Medicine, Daegu, Republic of Korea; 2 Institute for Cancer Research, Keimyung University, Daegu, Republic of Korea; 3 Institute of Medical Science, Keimyung University, Daegu, Republic of Korea; 4 Department of Pathology, Keimyung University Dongsan Medical Center, Daegu, Republic of Korea; 5 Department of Surgery, Keimyung University Dongsan Medical Center, Daegu, Republic of Korea; 6 Department of Radiation Oncology, Keimyung University School of Medicine, Daegu, Republic of Korea; 7 Department of Immunology, School of Medicine, Kyungpook National University, 680 Gukchaebosang-ro, Jung-gu, Daegu, 41944 Republic of Korea

**Keywords:** Cancer, Molecular biology

## Abstract

The expression profiles of conventional reference genes (RGs), including *ACTB* and *GAPDH,* used in quantitative real-time PCR (qPCR), vary depending on tissue types and environmental conditions. We searched for suitable RGs for qPCR to determine the response to radiotherapy in colorectal cancer (CRC) cell lines, organoids, and patient-derived tissues. Ten CRC cell lines (Caco-2, COLO 205, DLD-1, HCT116, HCT-15, HT-29, RKO, SW1116, SW480, and SW620) and organoids were selected and irradiated with 2, 10 or 21 grays (Gy) based on the previous related studies conducted over the last decade. The expression stability of 14 housekeeping genes (HKGs; *ACTB, B2M, G6PD, GAPDH, GUSB, HMBS, HPRT1, IPO8, PGK1, PPIA, TBP, TFRC, UBC*, and *YWHAZ)* after irradiation was evaluated using RefFinder using raw quantification cycle (Cq) values obtained from samples before and after irradiation. The expression stability of HKGs were also evaluated for paired fresh frozen tissues or formalin-fixed, paraffin-embedded samples obtained from CRC patients before and after chemoradiotherapy. The expression of *YWHAZ* and *TBP* encoding 14-3-3-zeta protein and TATA-binding protein were more stable than the other 12 HKGs in CRC cell lines, organoids, and patient-derived tissues after irradiation. The findings suggest that *YWHAZ* and *TBP* are potential RG candidates for normalizing qPCR results in CRC radiotherapy experiments.

## Introduction

Polymerase chain reaction (PCR) is a molecular biological technique that replicates and amplifies the desired region of a gene and is widely used to determine gene expression^1^. In particular, quantitative real-time PCR (qPCR) is considered the most accurate method and reliable tool for relative gene expression studies^2^. In qPCR, the expression data must be normalized with respect to another gene called reference gene (RG), which is expressed at a constant level regardless of the biological state, tissue types, and experimental conditions, to accurately interpret the expression of genes of interest^3^. Genes expressed universally at constant levels in all cells and tissues regardless of the conditions are called housekeeping genes (HKGs) and are generally constitutive genes that perform basic functions required for cellular existence. Representative HKGs such as *ACTB* and *GAPDH* have been used as RGs (also called normalizers, internal standards, internal controls, or reference standards) for qPCR^2^.

However, accumulating evidence shows that the expression of *ACTB* or *GAPDH* is altered by experimental conditions and should not be presumed to be suitable RGs for qPCR^4,5^. Furthermore, analyses of large RNA sequencing (RNA-seq) or real-time reverse transcription PCR datasets of various human tissues have demonstrated that the expression of *ACTB* and *GAPDH* vary significantly depending on tissue types and individuals^6,7^. Many unsuccessful efforts have been made to identify HKGs that are universally and constantly expressed regardless of study conditions, and some subscribe to the opinion that no universal HKG exists^8^. Rather, it has been suggested that using different RGs might be appropriate depending on experimental conditions (cell types, disease conditions, and intervention types). In addition, it has been strongly argued that conducting experiments to determine a suitable RG for each study condition should be seriously considered in advance^9^.

Globally, colorectal cancer (CRC) is the second leading cause of cancer death^10^ and is expected to become the leading cause of cancer-related deaths in the population aged 20 to 49 by 2030^11,12^. Radiation therapy is used to reduce CRC tumor mass by causing direct DNA damage or generating free radicals that induce tumor cell death^13^, in particular for the neoadjuvant treatment algorithm of locally advanced rectal cancer. Given its anatomic position and the risk to invade the pelvic wall, rectal cancer benefits from shrinkage prior to surgery to reduce the local relapse rate, and increase sphincter preservation and overall survival^14–16^. Recently, neoadjuvant radiotherapy in combination with immunotherapy has attracted attention as an investigational strategy for CRC treatment as it increases tumor antigen release and impairs mismatch^17,18^. Molecular characterizations to detect oncogenes such as *APC* and *KRAS*, which aid early diagnosis of CRC to determine whether targeted therapy can be applied and predict treatment prognosis, are mainly based on PCR estimations^19^. Furthermore, PCR experiments have been widely used to elucidate the mechanisms responsible for CRC cell death by radiotherapy, identify biomarkers, and predict radiotherapy responses in CRC patients^20–22^. These studies indicate the importance of RGs in CRC radiotherapy experiments. However, radiation induces DNA damage resulting in alteration of the expression of genes, including the HKGs, in human cells over time^23,24^. For example, in an analysis of a microarray dataset of 161 colon cancer specimens, *ACTB* showed lower stability than other HKGs^25^. Recently, Iyer et al. showed that the dose of ionizing radiation plays a crucial role in determining the stability of HKGs^26^. The study also suggested that a better estimate of normalizing gene expression in cancer cell lines such as head and neck, non-small cell lung, and pancreatic cancer cell lines after ionizing radiation treatment can be obtained using more than one HKG. Although several studies have been conducted to identify a suitable RG for CRC^5,25^, an RG suitable for PCR in CRC radiotherapy studies has not been reported.

The present study aimed to identify a stable RG suitable for qPCR in irradiated CRC cells or tissues. The expression stabilities of 14 well-known HKGs, including *ACTB* and *GAPDH*, were investigated by qPCR. The experimental conditions, including the CRC cell lines (Caco-2, COLO 205, DLD-1, HCT116, HCT-15, HT-29, RKO, SW1116, SW480, and SW620), irradiation doses (2 and 10 grays (Gy)), and exposure time (72 h), were determined by reviewing several related studies conducted over the last decade. Furthermore, we analyzed the expression stabilities of the HKGs against irradiation using CRC organoid, an ex vivo research model used in various cancer studies^27^. Finally, as gene expression after irradiation varies in vitro and in vivo due to the presence of cellular microenvironment^28^, we validated the expression stability of the HKGs using the fresh frozen tissues and formalin-fixed, paraffin-embedded (FFPE) samples obtained from patients with rectal cancer who underwent chemoradiotherapy (CRT).

## Results

### Selection of experimental conditions

**Figure 1:**
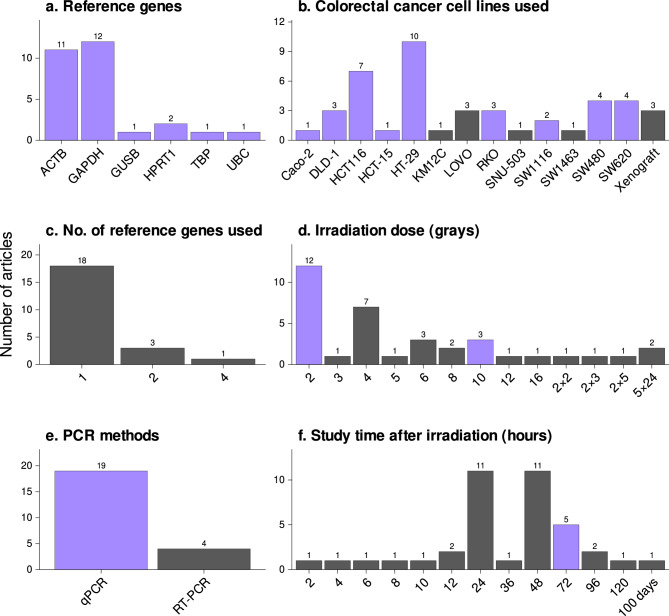
Experimental conditions frequently used in published studies reporting gene expression as determined using PCR in colorectal cancer (CRC) tissues after irradiation. The bar graphs show experimental settings frequently used in previous publications to study gene expression by PCR after irradiating CRC cells. Studies that have used more the one reference gene or CRC cell line were counted separately. Violet bars indicate experimental conditions selected for the present study.

In the present study, 22 CRC-related studies using PCR for gene expression analyses were identified from 242 CRC-related studies reviewed (Supplementary Figure S1 and Table S1). The most frequently used experimental conditions for PCR of CRC cell lines after irradiation are shown in Fig. 1. Six RGs, including *GAPDH*, *ACTB, HPRT1*, *GUSB*, *TBP*, and *UBC,* were in these studies for PCR normalization, of which *GAPDH* (12/22 studies) and *ACTB* (11/22 studies) were most frequently used. A single RG was used in most studies (18/22), wherein multiple RGs were used only in 4 of the 22 studies. HT-29, HCT116, SW480, and SW620 CRC cell lines were frequently used, and the most used irradiation doses were 2, 4, 6, and 10 Gy. Times used between irradiation and RNA isolation were 24, 48, and 72 h. Based on the experimental conditions used in 22 articles, we subjected ten CRC cell lines to irradiation. The ten CRC cell lines utilized in our study include Caco-2, COLO 205, DLD-1, HCT-15, HCT116, HT-29, RKO, SW1116, SW480, and SW620 (detailed information of these cell lines can be found in Supplementary Table S2 and S3). These cell lines were treated with 2 or 10 Gy of radiation and then incubated them for 72 h before RNA isolation (Fig. 1; violet color bars). In previous studies, RNA harvesting was mostly performed at 24 or 48 h after irradiation (Fig. 1f). However, in our preliminary experiments in CRC cell lines^18^, changes in cell morphology and increases in the Sub-G1 fraction were evident at 72 h after 10 Gy. Therefore, 72 h was selected in the present study.

### Expression stabilities of the HKGs in CRC cell lines

**Figure 2:**
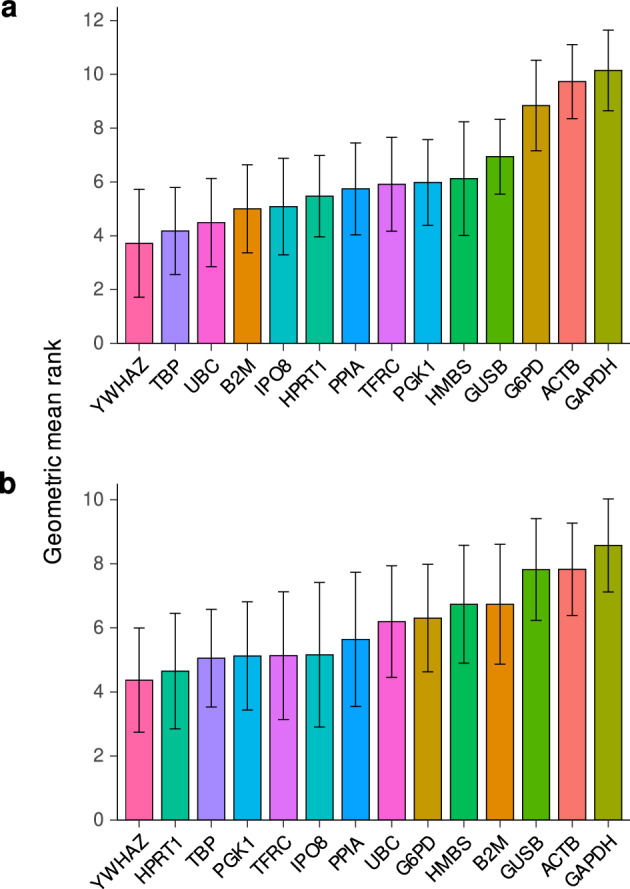
Gene expression stabilities in CRC cell lines exposed to radiation. The bar plots show combined results for the ten CRC cell lines. *YWHAZ*, and *TBP* are the most stably expressed genes after exposure to 2 (**a**) or 10 (**b**) grays of radiation. *ACTB* and *GAPDH* are relatively unstable. Colors represent specific genes, and error bars represent standard deviations.

After irradiating the ten CRC cell lines, we evaluated the expression stabilities of 14 candidate RGs including the 6 genes used in the 22 studies (Fig. 1) and 8 genes (*B2M*, *G6PD*, *HMBS*, *IPO8*, *PGK1*, *PPIA*, *TFRC*, and *YWHAZ*) recently studied for radiation stability in 6 different cancer cell lines, which included head and neck cancer, non-small cell lung cancer, and pancreatic cancer cell lines^26^. Detailed information on these genes is provided in Supplementary Table S4. Quantification cycle (Cq) values obtained by qPCR were used to estimate the stabilities of target gene expression by calculating the geometric means of stability rankings obtained using the four algorithms. The expression stability rankings of candidate RGs after irradiation in the ten CRC cell lines are shown in Fig. 2 and Supplementary Figure S2 and S3. The findings revealed that the stability of the genes varied with the radiation dose and cell lines. To comprehensively evaluate the expression stability across the different CRC cell lines, a combined geometric mean of the stability ranks was calculated. The analysis revealed that *YWHAZ* as the most stable gene after exposure to 2 and 10 Gy of radiation, followed by *TBP*. In contrast to several previous studies, we found that *ACTB* and *GAPDH*, the two most frequently used RGs for PCR normalization in the 22 articles, exhibited lower stabilities than the other genes examined. Furthermore, as irradiation kills cancer cells, we evaluated the levels of cell death in the ten CRC cell lines after each radiation dose. After irradiation at 2 or 10 Gy, 1.9% to 11.2% and 13.4% to 51.6% of the CRC cells, respectively, were in the sub-G1 fraction (Supplementary Figure S4).

### Expression stabilities of the HKGs in CRC organoids

**Figure 3:**
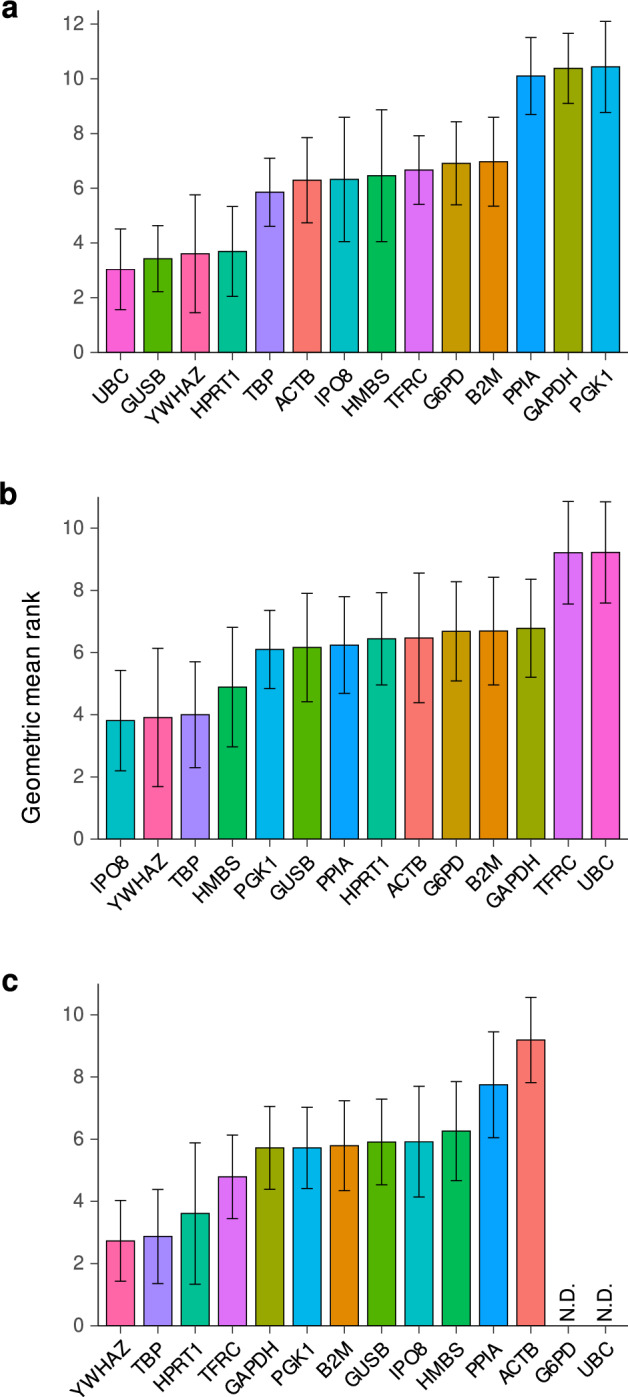
Gene expression stabilities in rectal cancer patient-derived samples exposed to radiation. Gene expression stabilities were analyzed in rectal cancer patient-derived samples including: (**a**) Organoids. (**b**) Fresh frozen samples. (**c**) Formalin-fixed, paraffin-embedded samples. *ACTB* and *GAPDH* are relatively unstable reference genes. The error bars represent standard deviations. N.D. indicates genes that were not detected in the analysis.

We repeated the above experiment using patient-derived CRC organoids. qPCR showed *UBC* and *GSUB* were the most stable genes at 21 Gy/3 fractions (Fig. 3a and Supplementary Figure S5). *YWHAZ* and *TBP*, which showed good overall stability in the cell line experiments, ranked 3rd and 5th, respectively, out of the 14 genes. *ACTB* and *GAPDH* were the less stable than *YWHAZ* and *TBP*, concordant with the cell line results. After irradiation with 21 Gy, three tested organoids demonstrated viabilities of 38.1%, 49.9%, 13.9% (Supplementary Table S5).

### Expression stabilities of the HKGs in patient-derive tissues

The stability of gene expression was evaluated in rectal cancer patients who underwent CRT using fresh frozen tissues and FFPE samples. *YWHAZ* and *TBP* demonstrated remarkable stability in gene expression throughout the CRT, securing the 2nd and 3rd positions in the fresh frozen tissues, and the 1st and 2nd positions in the FFPE samples, respectively (Fig. 3b and c and Supplementary Figure S6 and S7). Conversely, *ACTB* and *GAPDH* exhibited lower stability compared to *YWHAZ* and *TBP*. *G6PD* and *UBC* were not detected in certain FFPE samples (refer to Supplementary Figure S7).

### Statistical evaluation of expression stability

We conducted a permutational univariate analysis of variance and post hoc analysis to compare the stability of expression of each gene across the entire sample types (Table 1). *YWHAZ* exhibited significantly higher expression stability compared to *ACTB* and *GAPDH*. Specifically, the mean rank of *YWHAZ* was 5.18 and 5.41 lower than *ACTB* and *GAPDH*, respectively. Moreover, *YWHAZ* displayed significantly higher stability compared to *G6PD*, *GUSB*, *HMBS*, *PPIA*, and *TFRC*. Similarly, *TBP* demonstrated greater stability than both *ACTB* and *GAPDH*, with mean rank differences of −4.82 and −5.05, respectively. Additionally, in addition to *YWHAZ* and *TBP*, *HPRT1* and *IPO8* also exhibited statistically higher stability than *ACTB* and *GAPDH*.

**Table 1:** Post hoc comparisons of the gene expression stability ranks.

Genes	*ACTB*	*B2M*	*G6PD*	*GAPDH*	*GUSB*	*HMBS*	*HPRT1*	*IPO8*	*PGK1*	*PPIA*	*TBP*	*TFRC*	*UBC*
*B2M*	−2.08												
	(1.000)												
*G6PD*	−0.79	1.23											
	(1.000)	(1.000)											
*GAPDH*	0.23	2.31	1.01										
	(1.000)	(0.954)	(1.000)										
*GUSB*	−1.74	0.34	−0.90	−1.97									
	(1.000)	(1.000)	(1.000)	(1.000)									
*HMBS*	−1.88	0.20	−1.04	−2.11	−0.14								
	(1.000)	(1.000)	(1.000)	(1.000)	(1.000)								
*HPRT1*	−3.62	−1.54	−2.72	−3.85	−1.88	−1.74							
	(0.014)*	(1.000)	(0.298)	(0.005)*	(1.000)	(1.000)							
*IPO8*	−3.44	−1.36	−2.55	−3.67	−1.70	−1.56	0.18						
	(0.026)*	(1.000)	(0.492)	(0.011)*	(1.000)	(1.000)	(1.000)						
*PGK1*	−2.41	−0.33	−1.55	−2.64	−0.67	−0.53	1.21	1.03					
	(0.728)	(1.000)	(1.000)	(0.380)	(1.000)	(1.000)	(1.000)	(1.000)					
*PPIA*	−1.78	0.29	−0.94	−2.01	−0.04	0.10	1.83	1.66	0.62				
	(1.000)	(1.000)	(1.000)	(1.000)	(1.000)	(1.000)	(1.000)	(1.000)	(1.000)				
*TBP*	−4.82	−2.74	−3.88	−5.05	−3.07	−2.93	−1.20	−1.37	−2.41	−3.03			
	(< 0.001)*	(0.282)	(0.005)*	(< 0.001)*	(0.096)	(0.152)	(1.000)	(1.000)	(0.732)	(0.111)			
*TFRC*	−1.85	0.23	−1.01	−2.08	−0.11	0.03	1.77	1.59	0.56	−0.07	2.96		
	(1.000)	(1.000)	(1.000)	(1.000)	(1.000)	(1.000)	(1.000)	(1.000)	(1.000)	(1.000)	(0.138)		
*UBC*	−2.37	−0.35	−1.54	−2.59	−0.68	−0.55	1.14	0.97	−0.03	−0.64	2.30	−0.57	
	(0.811)	(1.000)	(1.000)	(0.435)	(1.000)	(1.000)	(1.000)	(1.000)	(1.000)	(1.000)	(0.980)	(1.000)	
*YWHAZ*	−5.18	−3.11	−4.24	−5.41	−3.44	−3.30	−1.57	−1.74	−2.78	−3.40	−0.37	−3.33	−2.66
	(< 0.001)*	(0.086)	(0.001)*	(< 0.001)*	(0.026)*	(0.043)*	(1.000)	(1.000)	(0.250)	(0.031)*	(1.000)	(0.039)*	(0.360)

After obtaining significant results from permutation-based ANOVA results for gene expression stability ranks among each house keeping genes (HKGs). We conducted post hoc comparisons using Dunn's test with Bonferroni correction to identify pairwise differences in ranks between HKGs. Each number in the table represents the differences in ranks of expression stability ranks between the genes listed in the respective column and row. The values inside parentheses indicate the Bonferroni-corrected p-values to control the family-wise error rate at α = 0.05. Genes that exhibited statistically significant differences in stability are denoted with an asterisk (*).

### Expression level of the HKGs in CRC cell lines, organoids, and patient tissues

**Figure 4:**
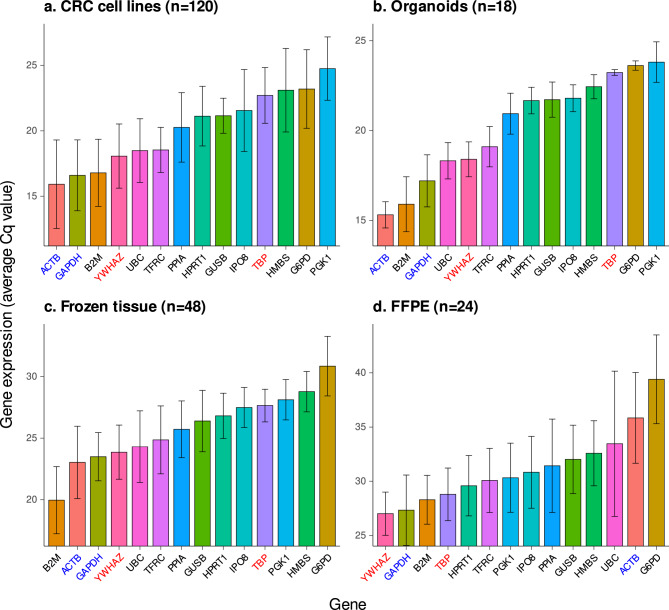
Gene expression levels of HKG in CRC cell lines or patient-derived samples. The average Cq values obtained from PCR analysis in (**a**) CRC cell lines, (**b**) organoids, (**c**) fresh frozen samples, (**d**) formalin-fixed, paraffin-embedded samples. The number of raw Cq values obtained is indicated by ‘n’. The error bars represent standard deviations.

To evaluate the relative expression levels of HKGs in various sample types, average Cq values were compared. *GAPDH* and *B2M* were consistently showed higher expression levels compared to other HKGs across all four sample types (Fig. 4). *ACTB* exhibited high expressed in three of the sample types, except in FFPE samples. *YWHAZ* showed higher expression level ranked 1st to 5th depending on the sample type. On the other hand, *TBP* consistently exhibited lower expression levels, ranking 10th to 11th in most sample types, except in FFPE samples where it ranked 4th. There findings were further supported by the RNA sequencing-based analysis, which demonstrated the consistent higher expression levels of *GAPDH*, *ACTB*, and *YWHAZ*, and the lower expression level of *TBP* (Supplementary Figure S8).

## Discussion

HKGs are ubiquitously expressed in all cell and tissue types and disease conditions regardless of treatments^8,29^. HKGs are commonly used as internal standards to normalize raw Cq values obtained using qPCR. However, the expression of HKGs may be dependent on experimental conditions, and thus, it was proposed that efforts be made to identify a case-specific RG for qPCR suitable for each experimentation^9^.

RT causes DNA damage and kills cancer cells, and gene expression in dying cells differs from that in living cells^24,26^. This study evaluated the expression stabilities of RGs commonly used to study the effect of irradiation on gene expression in CRC. According to “*The Minimum Information for Publication of Quantitative Real-Time PCR Experiments*” guideline published in 2009, qPCR experiments performed without critical evaluation of the RG used are poorly designed and difficult to reproduce^3^. We investigated the experimental conditions commonly used to study radiotherapy-induced changes in the expression of CRC-associated genes and estimated the expression stabilities of 14 HKGs commonly used as RGs in CRC radiotherapy studies, including *GAPDH* and *ACTB*. These genes are encoded by RNA polymerase II and represent diverse basic cellular pathways in normal cells, therefore, reducing the possibility of co-regulated genes that may induce potential artifacts in estimating the response to radiation therapy in heterogeneous cancer cells. Therefore, it has been suggested that stable HKGs that are unlikely to be co-regulated would be critical for normalization in radiation studies^26^. However, as these 14 HKGs have a broad range of Cq values, the most suitable RG for accurate target gene normalization depends on the expression level of the target gene. In this study, Tm values 59 °C and amplicon sizes of < 197 bp were ensured to minimize inter- and intra-variability during amplification and reduce the variability associated with PCR amplification efficiency, respectively^30^.

In this study, we evaluated the expression stability of the HKGs in ten widely used CRC cell lines. Furthermore, we verified the findings obtained using CRC cell lines in patient-derived organoids and tissues. Unlike CRC cell lines which form monolayers, CRC organoid cells are arranged in three dimensions, and as a result, cell exposure to nutrients and oxygen is variable^31^. Nonetheless, the organoids revealed consistent results as obtained using the CRC lines.

In this study, we applied different irradiation dosages for cell lines and organoids. For the cell lines, we selected 2 Gy, which corresponds to the one fraction dose in standard fractionated radiotherapy, on its common usage in cell line experiments. Additionally, based on our preliminary cell viability tests, we found that 10 Gy induced more than 50% cell death in many CRC cell lines. Consequently, we included 10 Gy as another dosage in this study. In contrast, our organoid experiment revealed that even 10 Gy did not result in noticeable cell death. To ensure a substantial impact on the organoids, we applied a higher dosage of 21 Gy. In general, the irradiation dosages of 2, 10, and 21 Gy are considered as medium, high, very high doses, respectively^32^.

Reportedly, the expression stabilities of HKGs and gene expression responses to radiotherapy differ in vitro and in vivo, which has been suggested to be due to the differences in endogenous cellular microenvironments^26^. Therefore, we evaluated the expression stability of the HKGs after radiotherapy in vivo in patient-derived tissues using fresh frozen tissues and FFPE samples. Surprisingly, despite differences in treatment regimens and the presence of complex microenvironments, the gene expression stability against irradiation in rectal cancer tissues in vivo closely paralleled the findings observed in CRC cell lines or organoids.

qPCR was performed using the conditions mentioned above to obtain raw Cq values, and these values were used to evaluate expression stabilities using four algorithms (Delta-Ct, GeNorm, BestKeeper, and NormFinder). Rankings of the expression stabilities of genes were obtained by calculating the geometric means of ranking values obtained using the four algorithms. Several studies have also adopted this method to find suitable RGs^33–35^.

Combined results for the ten CRC cell lines showed the expression of the *YWHAZ* and *TBP* genes was relatively less altered by irradiation at 2 or 10 Gy. However, rankings of gene expression stabilities depended on cell type and radiation dose. For example, at 2 Gy, *GAPDH* was the second most stably expressed gene in DLD-1 cells but one of the most unstable genes in other cell lines. Likewise, *GAPDH* was not stable at 10 Gy in DLD-1 cells. However, these differences in expression stability were independent of changes in cell viability after irradiation. Furthermore, the MSI status, mutation status, and the original cancer stage of the cell lines did not appear to be associated with the gene expression stability of HKGs after irradiation (Supplementary Table S2). These results show the expression stabilities of a given HKG under specific conditions are rarely predictable and support the suggestion that case-specific RGs should be used for normalization purposes^9^. In organoids, *YWHAZ* and *TBP* were more stably expressed than *ACTB* and *GAPDH*. Furthermore, the analyses of the fresh frozen tissues and FFPE samples consistently revealed *YWHAZ* and *TBP* as the most stable among the tested HKGs. On the other hand, *GAPDH* and *ACTB* showed relatively lower stability.

To sum up, *YWHAZ* and *TBP* showed good overall stability after radiotherapy under all study conditions, including the cell lines, organoids, and patient-derived tissues. However, the expression stabilities of the other genes, including *GAPDH* and *ACTB*, depended on the sample type and radiotherapy dose. Except for *YWHAZ* and *TBP*, the expression stabilities of these genes generally differed in vitro (in cell lines), ex vivo (in organoids) and in vivo (in fresh frozen tissues and FFPE samples). The variation in expression stabilities of the genes could be attributed to the experimental conditions— four different types of samples included in our study were exposed to different radiation doses, microenvironments, and cell heterogeneities. Nevertheless, *YWHAZ* and *TBP* were stably expressed irrespective of sample type or radiation dose, suggesting their potential suitability as reliable RGs in CRC radiotherapy studies. Recently, attempts have been made to use stably expressed genes instead of exogenous additions to remove batch effects between data sets during single-cell sequencing analysis^36^. Our findings indicate *YWHAZ* and *TBP* might be useful in studies other than qPCR studies.

*YWHAZ* encodes 14-3-3-zeta protein, a member of the highly conserved 14-3-3 family involved in essential cellular processes^37^. *TBP*, on the other hand, encodes the TATA-binding protein, which is the most conserved transcription initiation factor across bacteria to eukaryotes^38^. These two highly conserved genes have been reported to exhibit stable expression in various cells or tissues in numerous previous studies. In human lumbar intervertebral disc tissues, *TBP* has been identified as the most stably expressed gene, followed by *YWHAZ*^39^. *TBP* was also the most stable standalone gene for gene expression study for VEGF stimulation in human bone marrow-derived stromal cells and adipose-derived stromal cells^40^. The study showed that *TBP* plus *YWHAZ* was the best two-RG combination. *YWHAZ* has been suggested as a suitable RG for PCR normalization in MCF7, HCT116, and HepG2 cell lines^41^. In this study, TBP ranked 3rd in terms of gene expression stability among the 12 tested genes, including *ACTB* and *GAPDH*. Furthermore, *YWHAZ* has been reported to exhibit stable expression in long-term expanded human bone marrow-derived mesenchymal stem cells^42^, as well as adipose- derived mesenchymal stem cells and the tendon cells^43^. *TBP* was identified as the most stably expressed gene in bone marrow stromal cells obtained from patients with osteoarthritis and healthy donors^44^. The stability of *TBP* expression after irradiation has been observed in head and neck cancer and pancreatic cancer cell lines^26^. Moreover, *TBP* has already been used as an RG in several studies on prostate cancer^45,46^, and in the lymph nodes of a prostate cancer patient, *TBP* and *HPRT1* showed good expression stabilities^47^. On the other hand, it should be noted that *TBP* has been reported to be unstable under hypoxic conditions in human cardiac stem cells^48^, This suggests that certain genes may nor serve as universal RG across different experimental conditions. Therefore, *YWHAZ* and *TBP* are only suitable as RGs in radiotherapy studies on CRC cell lines or rectal cancer tissues, and their expression stability needs to be confirmed on an individual basis when experimental settings differ.

Like most other human genes, *TBP* has more than one transcript isoform, and therefore, alternative splicing of *TBP* as an RG should be addressed, as was previously recommended^2,6^. According to the NCBI *Homo sapiens* Annotation Release 109, *TBP* has two isoforms. *TBP-1* and *TBP-2* have transcript lengths of 1857 and 1655 bp that differ in their 5’ UTRs and coding sequences, and consequently, *TBP-2* has a shorter N-terminus. To investigate the expression stabilities of these *TBP* isoforms for CRC radiotherapy studies, we designed *TBP-1* isoform-specific primers and performed cell line experiments (Supplementary Table S8). We found the expression stability of *TBP-1* was not significantly different in CRC cell lines from that observed in a previous experiment using *TBP* primers non-specific for *TBP-1* or *TBP-2* (Supplementary Figure S9).

*YWHAZ* showed higher expression levels compared to the other tested HKGs, while *TBP* exhibited lower expression levels (Supplementary Figure S8). For reliable normalization of PCR results, it is important to select RGs that have similar expression levels to the genes of interest. In this context, researchers conducting studies on CRC and radiotherapy need to consider the expression level of target genes when choosing *YWHAZ* and *TBP* as a single RG. Using a combination of the multiple RGs has proven to be more reliable than using a single RG for PCR normalization^49,50^. Furthermore, *YWHAZ* and *TBP* together can cover a wide range of target gene expression level. Therefore, we highly recommend using these two most suitable RGs, *YWHAZ* and *TBP*, in combination for irradiation studies in CRC.

The findings of this study have to be seen in light of some limitations. Specifically, we were unable to detect *G6PD* and *UBC* genes in some of the FFPE samples, which may be due to nucleic acid degradation. Notably, three samples lacking *G6PD* or *UBC* genes exhibited DV200 values of 51.41, 38.87, and 67.82 (Supplementary Table S7). Furthermore, the gene expression analysis of FFPE samples revealed contrasting results compared to cell lines, organoids, and fresh frozen tissues. *ACTB* showed relatively lower expression, while *TBP* exhibited higher expression in FFPE samples. Previous research on gene expression analysis using FFPE samples from CRC patients emphasized the advantages of using short amplicons for gene expression analysis in these samples. They proved that PCR using short amplicon was less susceptible to nucleic acid degradation in FFPE samples compared to long amplicons. Given the significance of FFPE samples in translational medical research, further investigations are necessary to achieve optimal primer designs for RGs with shorter amplicons that could improve PCR efficiency for FFPE samples.

In conclusion, this study demonstrates that the expression of *YWHAZ* and *TBP* are not substantially altered by irradiation in CRC cell lines or tissues. It suggests that when analyzing radiotherapy-induced changes in gene expression in CRC using qPCR, *YWHAZ* and *TBP* are preferentially considered an internal control gene for normalization purposes. The findings of this study will serve as a foundation for preclinical studies for the normalization of expression data of genes to evaluate the response to radiation therapy not only in CRC but also in other cancers.

## Materials and methods

### Experimental conditions used in previous studies

We searched the PubMed database for frequently used experimental settings in previous CRC and radiotherapy studies published from January 1, 2010 to March 8, 2020. We obtained a total of 242 studies that included 'colorectal cancer' in the title and simultaneously included 'radiation' (n = 123), ‘radiotherapy’ (n = 99), or 'radiation therapy' (n = 20). Of these articles, 22 radiotherapy-related experimental studies investigated gene expression using PCR (Supplementary Table S1). We then investigated the RGs used for normalization of PCR data, irradiation doses, times to RNA isolation after irradiation, numbers of RGs, PCR methods, and CRC cell types used in these studies.

Clinical trials which involving radiotherapy were excluded. Of these articles, 22 radiotherapy-related experimental studies investigated gene expression using PCR.

### Irradiation of CRC cell lines

We included a total of 10 CRC cell lines, consisting of 9 previously used CRC cell lines sourced from the literatures, and one additional CRC cell lines, COLO 205 (Fig. 1b and Supplementary Table S2). Based on the review, the radiation doses were determined to be 2 or 10 Gy, and RNA was isolated 72 h after irradiation. All ten CRC cell lines used in this study were obtained from Korean Cell Line Bank (Korean Cell Line Research Foundation, Seoul, Korea) or ATCC (Rockville, MD, USA). Upon thawing the cryopreserved cell lines, they were subcultured 2–3 times in GIBCO RPMI 1640 medium or MEM (Thermo Fisher Scientific, Wilmington, DE, USA) supplemented with 10% heat-inactivated fetal bovine serum (Thermo Fisher Scientific), 2 mM L-glutamine, 100 μg/mL streptomycin, and 100 μg/mL penicillin. The detailed composition of the culture media is provided in Supplementary Table S3. Prior to conducting the irradiation experiments, the cellular morphology of each CRC cell lines was carefully examined and compared to the reference photos provided by the Korean Cell Line Bank. To estimate responses to irradiation, each CRC cell line was seeded in 6-well plates with predetermined cell numbers as indicated in Supplementary Table S3. Subsequently, the cells were incubated in a humidified atmosphere containing 5% CO_2_ at 37 °C. Cells were irradiated with 2 or 10 Gy of X-ray radiation in one fraction (6 megavolts; dose rate 200 cGy/s) using a linear accelerator (VitalBeam; Varian Medical Systems, Palo Alto, CA, USA). After irradiation, culture media were replaced with a fresh medium to exclude irradiation effects on the culture medium. Total cellular RNA was extracted 72 h after irradiation. To assess the extent of cell death after irradiation, we performed cell counting, flow cytometry, and XTT assays. Cell counting was carried out using the LUNA-FL™ Automated Cell Counter (Logos Biosystems, Anyang, South Korea). For flow cytometry analysis, cells were stained with propidium iodide and analyzed using FACSCanto II (BD Biosciences, Franklin Lakes, NJ, USA). XTT assays were performed using WelCount™ following the manufacturer’s protocol (WelGENE, Gyeongsan, South Korea). Non-irradiated cells maintained under the same conditions were used as controls.

### Irradiation of patient-derived CRC organoids

Primary cancer cells were isolated from fresh tumor tissue samples obtained from three patients diagnosed with stage II CRC (Supplementary Table S5) using Gentle Cell Dissociation Reagent (STEMCELL Technologies, Vancouver, Canada). Cells were resuspended in DMEM/F12 (STEMCELL Technologies) supplemented with 1% bovine serum albumin and Matrigel (growth factor reduced, phenol red-free, Corning, NY, USA) and cultured in IntestiCult™ Organoid Growth Medium (STEMCELL Technologies) at 37 °C in 5% CO_2_. CRC organoids were irradiated with 21 Gy of X-ray radiation in three fractions in the same manner as described for the CRC cell lines. Afterward, culture media were replaced with a fresh medium after irradiation to eliminate the effects of radiation. Total cellular RNA was extracted after 72 h of incubation. Viability of organoids after irradiation was evaluate using the CellTiter-Glo® (Promega, Madison, WI, USA). Non-irradiated CRC organoids maintained under identical conditions were used as controls.

### RNA isolation from cell lines and organoids

After 72 h of irradiation, total cellular RNA from CRC cell lines or organoids (irradiated or non-irradiated) was extracted using TRI Reagent® (Molecular Research Center, Inc., Cincinnati, OH, USA). RNA was quantified using a NanoDrop 1000 (Thermo Fisher Scientific).

### RNA isolation from fresh frozen tissues and FFPE samples

Paired fresh frozen tumor tissues and FFPE samples were collected from eight and four patients with rectal cancer, respectively, both before and after undergoing CRT (Supplementary Table S6 and S7). These patients were diagnosed with rectal adenocarcinoma and underwent neoadjuvant concurrent CRT and primary tumor resection between August 2016 and December 2017. All patients received conventional long-course neoadjuvant CRT [five cycles of 5-fluorouracil-based (5-FU) chemotherapy and 50.4 Gy radiation]. Total mesorectal excision was performed within 6–8 weeks of CRT completion.

Total RNA was extracted from eight pairs of fresh frozen tumor tissues using QIAzol® Lysis Reagent, TissueLyser LT, and the RNeasy® Mini Kit (QIAGEN, Hulsterweg, Netherlands). Mineral Oil (Sigma-Aldrich, St. Louis MO, USA) and RNeasy® Mini Kit were used for the extraction of RNA from four pairs of FFPE samples. The quantity of extracted RNA determined using Quant-it™ RiboGreen RNA Assay method (Invitrogen, Waltham, MA, USA), employing VICTOR® Nivo Multimode Microplate Reader (PerkinElmer, Waltham, MA, USA). Furthermore, the quality of the extracted RNA was assessed using DV200 values measured with the 4200 TapeStation (Agilent Technologies, Santa Clara, CA, USA).

### Real-time PCR

cDNA was synthesized from 2 μg total RNA using MMLV reverse transcriptase (Promega) according to the manufacturer’s protocol. qPCR was performed on a LightCycler 480 real-time PCR system (Roche Diagnostics, Mannheim, Germany) using the HKG-specific primer pairs described in Supplementary Table S4 and SYBR Green Premix (Toyobo, Japan). We followed MIQE guidelines for real-time PCR^3^. A total of 14 candidate HKGs were evaluated. These genes were selected using the following criteria: expressed throughout the body and reflect diverse cellular functions. All selected genes were confirmed to be ubiquitously expressed throughout the body through the Illumina Human Body Map 2.0 (data accessible at NCBI GEO database, accession GSE30611) or Human Protein Atlas project^51^. These genes included structural and immune-related genes that play various cellular functions, such as metabolic enzymes and kinases involved in signaling pathways or transcription. Primers were designed to have a uniform annealing temperature (Tm) of 59 °C and an amplicon size of < 197 bp to reduce PCR amplification efficiency-induced variability^30^ using Primer3Plus^52^. Gene relative abundances were determined from raw Cq values. Three technical replicates were used in each experiment.

### Expression stabilities of the candidate HKGs

After obtaining Cq values of irradiated and non-irradiated samples by qPCR, RefFinder^53^ which utilizes four different evaluation algorithms (comparative delta-Ct^54^, GeNorm^50^, BestKeeper^55^, and NormFinder^25^) was used to evaluate the expression stabilities of the selected genes. Of these four algorithms, the comparative delta-Ct method compares the delta-Ct values of RG pairs in each sample for all possible RG combinations. Then, a gene with a smaller delta-Ct value deviation from other genes is considered more stably expressed. GeNorm uses a measure of gene expression stability, M, as the average pair-wise variation of a specific gene with all other tested genes. The gene with the lowest M values is the most stably expressed. BestKeeper measures the correlation coefficient between the Ct value of candidate RGs and the BestKeeper index calculated as the geometric mean of the Ct values of the HKGs with low standard deviation. Finally, NormFinder estimates expression variability in candidate RGs using a model-based approach that accounts for inter- and intra-group variability. To comprehensively evaluate the expression stabilities of the candidate HKGs, a geometric mean was calculated using rank values derived from four algorithms for each experimental setting. Subsequently, a combined geometric mean was computed by integrating the ranks across different CRC cell types and biological replicates. Missing Cq values were replaced with geometric mean Cq values of two qPCR replicates^50^.

### Statistical evaluation of expression stability

To compare the stability rank values among different genes, we conducted a one-way permutational univariate analysis of variance (ANOVA) using geometric mean ranks across the entire sample, including thirty-five geometric rank values for each HKGs. These values were collected from 10 CRC cell lines (20 ranking values from each 2 and 10 Gy irradiation), 3 organoids, 8 fresh frozen tissues, and 4 FFPE samples. We employed 1,000 permutations. Following the significant ANOVA result, we conducted post hoc tests using Dunn's test to identify specific groups that differed significantly from each other. To account for multiple comparisons, we applied Bonferroni correction to adjust the p-values. Statistical significance was determined at a threshold of adjusted P < 0.05. The statistical analyses were conducted using the R statistical software^56^.

### Expression level of HKGs in colon tissues: RNA sequencing-based analysis

RNA sequencing data obtained for paired tumor tissues before and after CRT from 11 patients with rectal cancer were utilized to assess the expression level of candidate HKGs. Detailed patient information and a comprehensive description of the RNA sequencing data acquisition method can be found in the previous article^18^. In summary, RNA sequencing was performed on a NextSeq500 sequencer (Illumina, San Diego, CA, USA), and count normalization was conducted using DESeq2^57^.

To further investigate the expression levels of HKGs in CRC tissues and adjacent normal colon tissues, the TCGA-COAD dataset was utilized. The raw RNA sequencing counts from TCGA-COAD were obtained using the TCGAbiolinks package^58^ in R, and count normalization was performed using DESeq2.

In order to evaluate the expression levels of HKGs in normal colon tissues, the expression data of the selected genes in the 5 normal colon samples were obtained from the Human Protein Atlas RNA-seq normal tissue dataset (www.proteinatlas.org; last accessed May 24, 2021).

### Ethics declarations

All methods were performed in accordance with the relevant guidelines and regulations. The present study was approved by the Institutional Review Boards of Keimyung University (IRB Nos. 2019-09-021) and the Dongsan Medical Center (2019-12-047). Prior to specimen collection, written informed consent was obtained from all participating patients.

### Supplementary Information

Supplementary Information.

## Data Availability

The raw RNA sequencing data and associated metadata for the patients have been deposited in the NCBI Gene Expression Omnibus (Accession Number: GSE233517).
