# Supplementary material for: YWHAZ and TBP are potential reference gene candidates for qPCR analysis of response to radiation therapy in colorectal cancer
Source: Sci Rep. 2023 Aug 9;13:12902. doi: 10.1038/s41598-023-39488-6 (PMC10412564; doi:10.1038/s41598-023-39488-6)
Supplement: Supplementary file 1 — Supplementary Information. [file 41598_2023_39488_MOESM1_ESM.pdf]

Supplementary Information

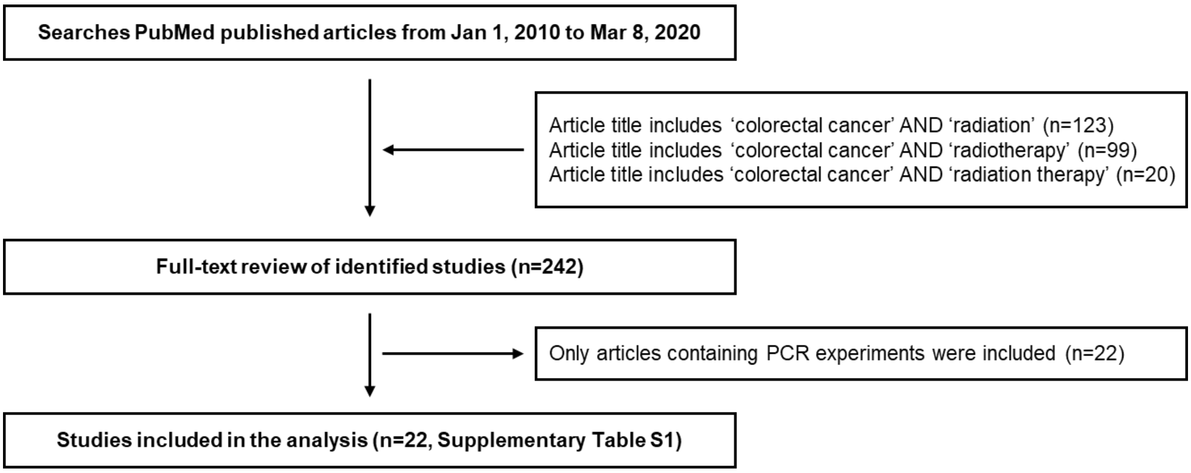

Supplementary Figure S1. Flow chart of literature search.

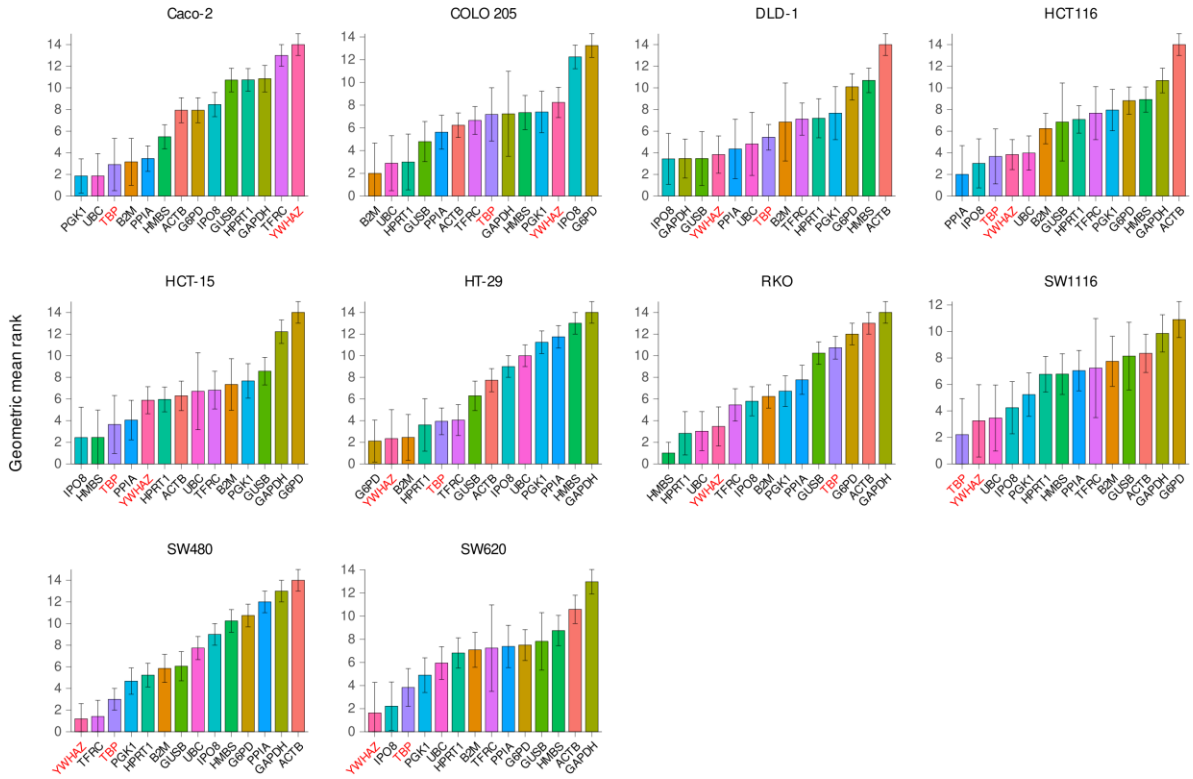

Supplementary Figure S2. Gene expression stabilities in ten CRC cell lines exposed to 2 Gy radiation. Colors represent specific genes, and error bars represent standard deviations.

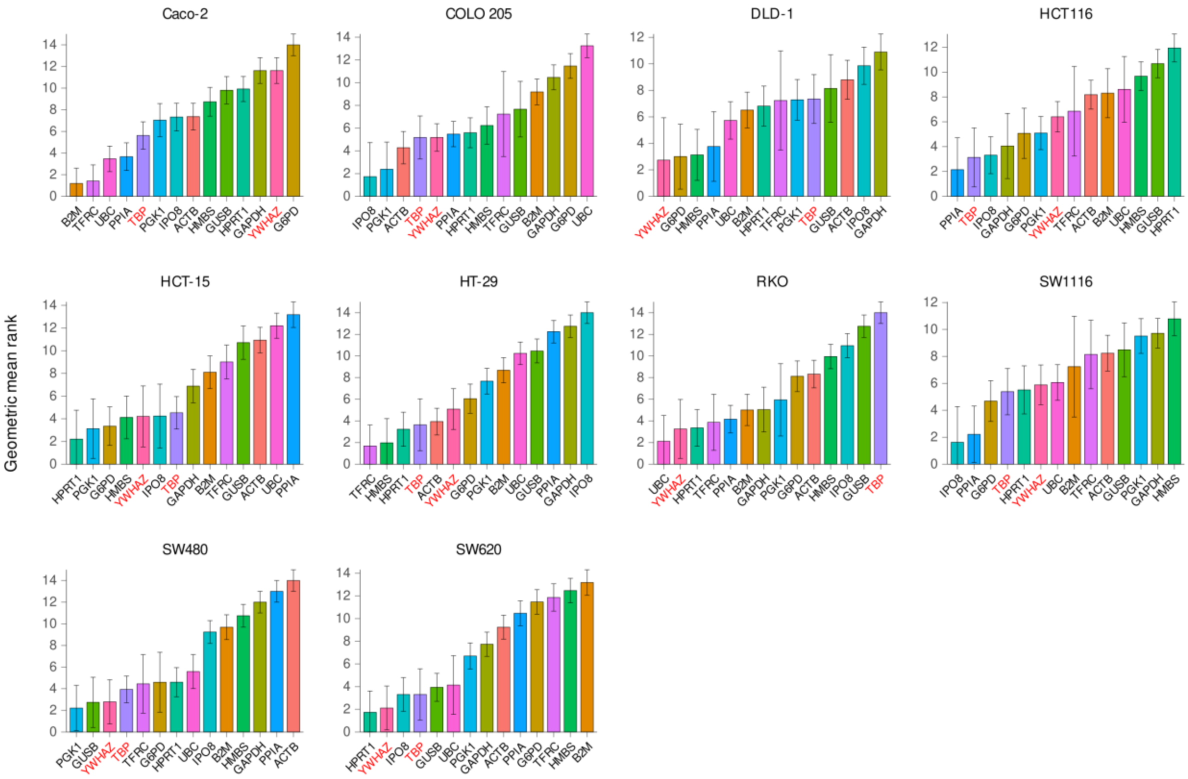

Supplementary Figure S3. Gene expression stabilities in ten CRC cell lines exposed to 10 Gy radiation. Colors represent specific genes, and error bars represent standard deviations.

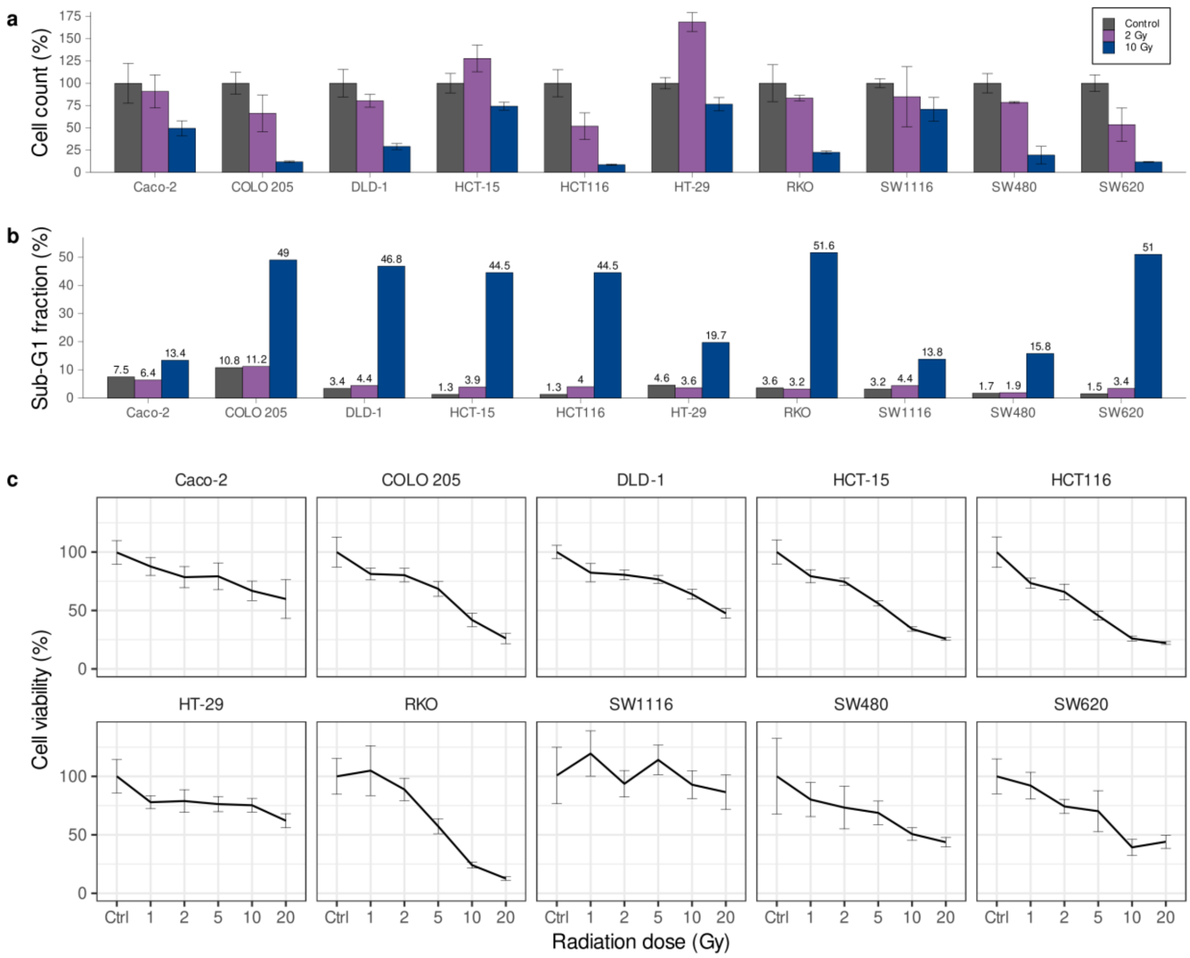

Supplementary Figure S4. Viability of CRC cell lines following irradiation. (**a**) Cell viability was assessed through cell counting. (**b**) Flow cytometry analysis determined the percentage of cells in sub-G1 fractions, indicating apoptotic cells after irradiation. (**c**) Cell viability was also evaluated using XTT assays. Error bars represent standard deviations.

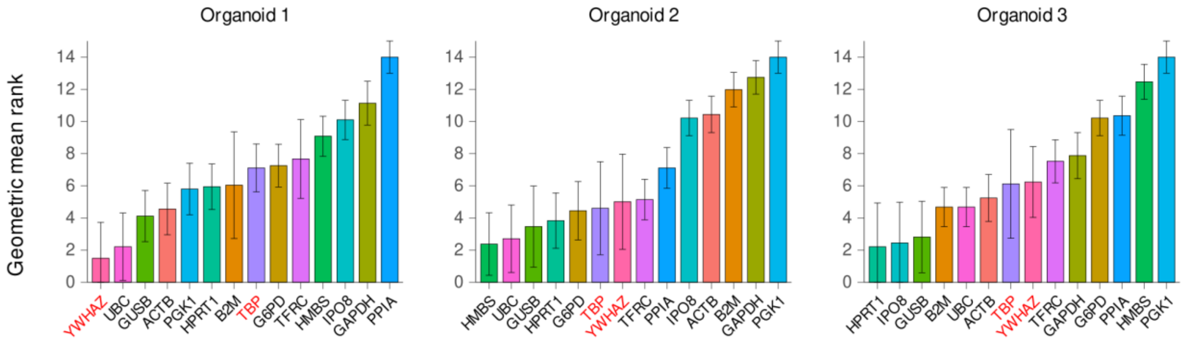

Supplementary Figure S5. Gene expression stabilities in three CRC patient-derived organoids exposed to 21 Gy radiation. Colors represent specific genes, and error bars represent standard deviations.

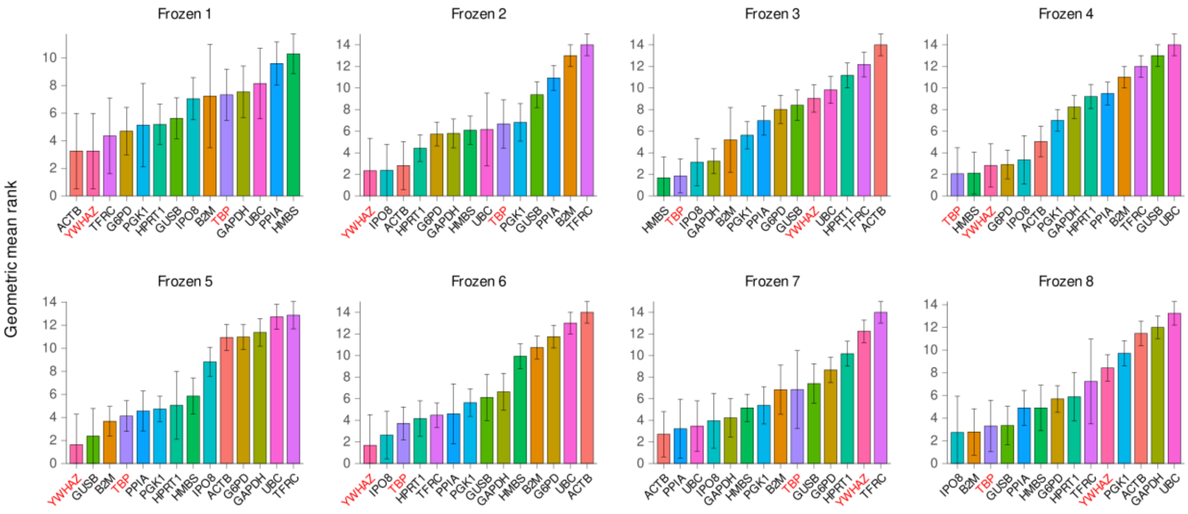

Supplementary Figure S6. Gene expression stabilities before and after radiotherapy in eight CRC patient-derived fresh frozen tissues. Colors represent specific genes, and error bars represent standard deviations.

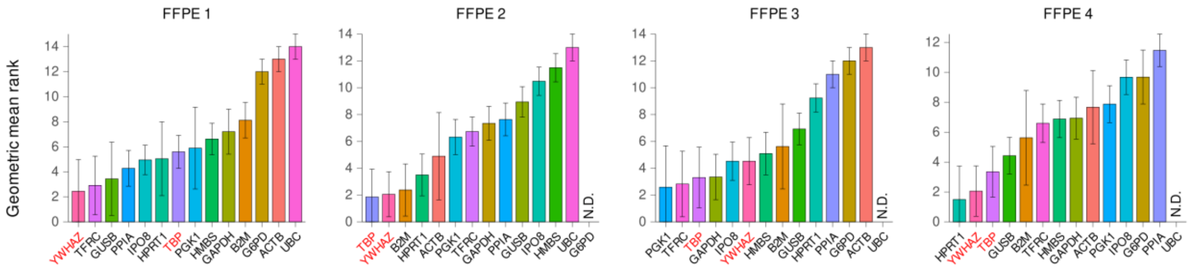

Supplementary Figure S7. Gene expression stabilities before and after radiotherapy in four CRC patient-derived formalin-fixed, paraffin-embedded samples. Colors represent specific genes, and error bars represent standard deviations. N.D., not detected.

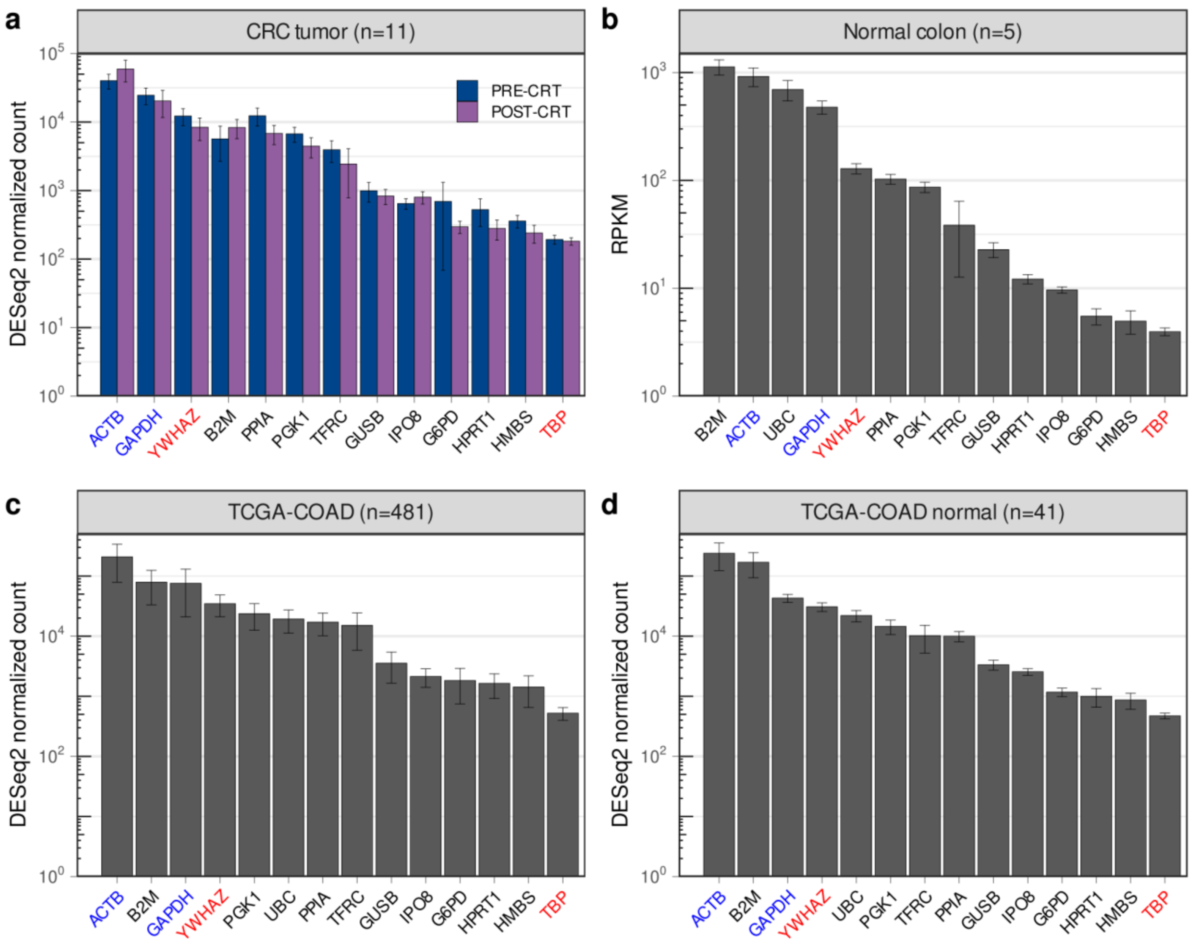

Supplementary Figure S8. Expressions of HKGs in tumor and normal tissues determined by RNA sequencing. (**a**) Gene expression analysis using RNA sequencing data (GSE233517). PRE-CRT and POST-CRT represent samples obtained before and after CRT, respectively. (**b**) Gene expression analysis in normal colon tissues obtained from Human Protein Atlas. Gene expression analysis of HKGs in both (**c**) tumor tissues and (**d**) adjacent normal tissues from the TCGA-COAD dataset. The number of cases is indicated by 'n'. The error bars represent standard deviations. RPKM, reads per kilobase of transcript per million mapped reads.

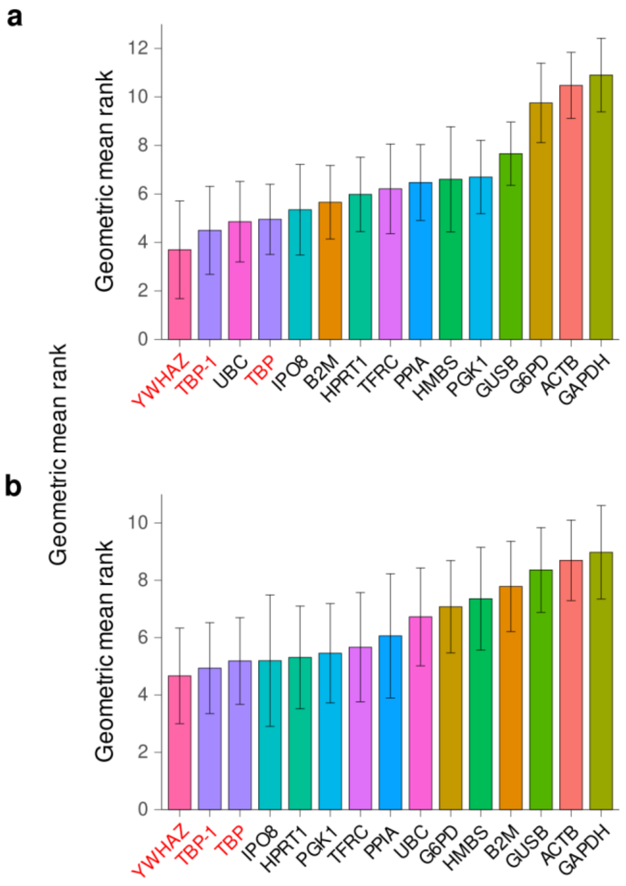

Supplementary Figure S9. Gene expression stability of the TBP-1 isoform in irradiated CRC lines. **(a)** and **(b)** show combined results for the ten CRC cell lines after exposure to 2-Gy or 10-Gy radiation, respectively. Error bars represent standard deviations.

Supplementary Table S1. Studies included in the analysis

No	Article title	Radiation dose (Gy)	PCR time after exposure (hr)	Reference gene	Method	Cell type used	Notes
1	A novel nanoparticle-based theranostic agent targeting LRP-1 enhances the efficacy of neoadjuvant radiotherapy in colorectal cancer	2	24	Not specified	qPCR	Xenograft (patient-derived)	This article does not describe which gene was used as the reference gene.
2	Antiproliferative and pro-apoptotic effects of *Cyclocarya paliurus* polysaccharide and X-ray irradiation combination on SW480 colorectal cancer cells	8	48	*GAPDH*	qPCR	SW480	
3	CDC25A, VAV1, TP73, BRCA1 and ZAP70 gene overexpression correlates with radiation response in colorectal cancer	16	12,24,48,72	*ACTB*	qPCR	SW620	An *Oryza sativa* sequence was used as a negative control.
4	Chemotherapy and radiotherapy downregulate the activity and expression of DNA methyltransferase and enhance Bcl-2/E1B-19-kDa interacting protein-3-induced apoptosis in human colorectal cancer cells	2, 4	48	*ACTB*	RT-PCR	SW480	
5	Epigallocatechin-3-gallate enhances radiation sensitivity in colorectal cancer cells through Nrf2 activation and autophagy	2	48	*GAPDH*	qPCR	HCT116	
6	Epsilon-Globin HBE1 enhances radiotherapy resistance by down-regulating BCL11A in colorectal cancer cells	5*24d	Not specified	*ACTB*	qPCR	HT-29, RKO, SW480, SW620	RT-PCR and qPCR used different reference genes.
	Epsilon-Globin HBE1 enhances radiotherapy resistance by down-regulating BCL11A in colorectal cancer cells	5*24d	Not specified	*GAPDH*	RT-PCR	Caco-2, DLD-1, HCT116, HT-29, KM12C, RKO, SW480,SW620	
7	Fibroblast growth factor receptor 4 induced resistance to radiation therapy in colorectal cancer	2, 4, 6	24	*GAPDH*	qPCR	HT-29	
8	Gefitinib enhances the effects of combined radiotherapy and 5-fluorouracil in a colorectal cancer cell line	2*1d, 2*2d, 2*3d	24,48,72	*ACTB, HPRT1*	qPCR	LOVO	RT was given in combination with chemotherapy. The A549 lung cancer cell line was used as a calibrator. This article does not show PCR experiment result.
9	Interferon-Induced IDO1 mediates radiation resistance and is a therapeutic target in colorectal cancer	6	48	*ACTB, GAPDH*	qPCR	DLD-1, HCT116, HT-29, xenograft(HCT116)	
10	Intricate macrophage-colorectal cancer cell communication in response to radiation	2*5d	6	*ACTB*	qPCR	RKO, SW1463	
11	LincRNA-p21 enhances the sensitivity of radiotherapy for Human colorectal cancer by targeting the Wnt/β-catenin signaling pathway	4	Not specified	*GAPDH*	qPCR	LOVO, SW1116	
12	Microenvironment and dose-delivery-dependent response after exposure to ionizing radiation in human colorectal cancer cell lines	2, 10, 2*5d	4	*HPRT1*	qPCR	DLD-1, HT-29	
13	MicroRNA-451a regulates colorectal cancer proliferation in response to radiation	2, 5, 10	24	*ACTB, GAPDH*	qPCR	HCT116	
14	Modulating survivin as a radioresistant factor, caspase-3, and apoptosis by omega-3 docosahexaenoic acid sensitizes mutant-p53 colorectal cancer cells to γ-irradiation	2, 10	48	*GAPDH*	RT-PCR	HT-29	
15	MTERFD1 promotes cell growth and irradiation resistance in colorectal cancer by upregulating interleukin-6 and interleukin-11	2, 4, 8	4,8,24,48	*GAPDH*	qPCR	HT-29, SW1116	
16	Radiation promotes colorectal cancer initiation and progression by inducing senescence-associated inflammatory responses	2	100d	*UBC, TBP, GUSB, GAPDH*	qPCR	Tissue (CDX2 APC flox/+ (CPC:Apc) mice)	Gene expression levels were normalized to the geometric mean of UBC, TBP, GUSB, and GAPDH
17	Radiation-induced DNA damage response and resistance in colorectal cancer stem-like cells	2, 4, 8	24,48	*ACTB*	qPCR	HCT116, HCT-15	
18	RBBP6 increases radioresistance and serves as a therapeutic target for preoperative radiotherapy in colorectal cancer	10	12,24,36	*GAPDH*	qPCR	HT-29, SW620	
19	Resistance of colorectal cancer cells to radiation and 5-FU is associated with MELK expression	4	24,48,72,96	*ACTB*	RT-PCR	SNU-503	
20	STING-dependent interferon-λ1 induction in HT29 cells, a human colorectal cancer cell line, after gamma-radiation	2, 3, 6, 12	24,72,120	*ACTB*	qPCR	HCT116, HT-29, LOVO	
21	Targeting the endoplasmic reticulum mediates radiation sensitivity in colorectal cancer	2	24,48,72,96	*ACTB*	qPCR	HT-29	
22	The long noncoding RNA-ROR promotes the resistance of radiotherapy for human colorectal cancer cells by targeting the p53/miR-145 pathway	Not specified	Not specified	*GAPDH*	qPCR	HCT116	

Supplementary Table S2. Human colorectal cancer cell lines used in this study

Cell lines	Morphology[1]	Tissue[1]	Disease[1]	Growth properties[1]	Dukes' Stage[1,2]	Derived from[1]	Age[1]	Ethnicity[1]	Gender[1]	MSI status[2,3]	CIMP[3]	CIN[2]	TP53[2,3]	KRAS[2,3]	BRAF[2,3]	PIK3CA[3]	PTEN[3]	SF2[4]	SF2[5]	SF5[5]	SF8[5]
Caco-2	Epithelial-like	Colon	Adenoca.	Adherent			72	White	Male	MSS	+	+	E204X	wt	wt	wt	wt				
COLO 205	Epithelial-like	Colon	Adenoca.	Mixed; adherent, suspension	D		70	White	Male	MSS	+		Y107fs Y103fs	wt	V600E	wt	wt	0.69	0.0048	0.0011	0.0003
DLD-1	Epithelial	Colon	Adenoca.	Adherent	C		Adult		Male	MSI	+	-	S241F	G13D	wt	E545K D549N	wt				
HCT-15	Epithelial	Colon	Adenoca.	Adherent	C				Male	MSI	+	-	S241F	G13D	wt	E545K D549N	wt	0.4	0.659	0.338	0.2006
HCT116	Epithelial	Colon ascendens	Carcinoma	Adherent	C	Primary tumor	48		Male	MSI	+	-	wt	G13D	wt	H1047R	wt	0.38	0.465	0.028	0.0019
HT-29	Epithelial	Colon	Adenoca.	Adherent		Primary tumor	44	White	Female	MSS	+	+	R273H	wt	V600E T119S	wt	wt	0.79	0.74	0.5	0.332
RKO	Epithelial	Colon	Carcinoma	Adherent		Primary tumor				MSI	+	-	wt	wt	V600E	H1047R	wt				
SW1116	Epithelial	Colon	Adenoca.	Adherent	A		73	White	Male	MSS	-	+	A159D	G12A	wt	wt	wt				
SW480	Epithelial	Colon	Adenoca.	Adherent	B	Primary tumor	50	White	Male	MSS	-	+	R273H P309S	G12V	wt	wt	wt				
SW620	Epithelial	Colon	Adenoca.	Adherent	C	Lymph node metastasis	51	White	Male	MSS	-	+	R273H P309S	G12V	wt	wt	wt	0.62	0.58	0.0758	0.000251

MSI, microsatellite instability; CIMP, CpG island methylator phenotype; CIN, chromosomal instability; SF2, surviving fractions of the cells after 2 Gy irradiation; Adenoca., adenocarcinoma; MSS, microsatellite stable; wt, wild type.

[References]

[1]American Type Culture Collection (ATCC): https://www.atcc.org/

[2]Ahmed, D. *et al.* Epigenetic and genetic features of 24 colon cancer cell lines. *Oncogenesis* **2,** e71–e71 (2013).

[3]Berg, K. C. G. *et al.* Multi-omics of 34 colorectal cancer cell lines - a resource for biomedical studies. *Molecular Cancer* **16,** 116 (2017).

[4]Torres-Roca, J. F. *et al.* Prediction of radiation sensitivity using a gene expression classifier. *Cancer Res* **65,** 7169–7176 (2005).

[5]Amundson, S. A. *et al.* Integrating global gene expression and radiation survival parameters across the 60 cell lines of the National Cancer Institute Anticancer Drug Screen. *Cancer Res* **68,** 415–424 (2008).

Supplementary Table S3. Culture conditions for the colorectal cancer cell lines and source of origin

Cell lines	Source	KCLB or ATCC catalog number	Passage number at acquisition	Culture media composition	Cell seeding count in 6-well plate (cells/well)
Caco-2	KCLB	30037.1	30	MEM (25mM HEPES, $NaHCO_3$-sodium bicarbonate)	2×10^5
COLO 205	KCLB	10222	25	RPMI 1640 (10%FBS, 2% P/S)	2×10^5
DLD-1	KCLB	10221	44	RPMI 1640 (10%FBS, 1% P/S)	1.5×10^5
HCT-15	KCLB	10225	45	RPMI 1640 (10%FBS, 2% P/S)	1×10^5
HCT116	KCLB	10247	37	RPMI 1640 (10%FBS, 1% P/S)	1×10^5
HT-29	KCLB	30038	39	RPMI 1640 (10%FBS, 1% P/S)	1×10^5
RKO	ATCC	CRL-2577	Unknown	Eagle's MEM (10%FBS, 1% P/S)	1×10^5
SW1116	KCLB	10233	57	RPMI 1640 (10%FBS, 2% P/S)	2×10^5
SW480	KCLB	10228	119	RPMI 1640 (10%FBS, 1% P/S)	1.5×10^5
SW620	KCLB	60068	8	RPMI 1640 (10%FBS, 3% P/S)	1.5×10^5

KCLB, Korea Cell Line Bank; ATCC, American Type Culture Collection; MEM, minimum essential media; RPMI, Roswell Park Memorial Institute media; FBS, fetal bovine serum; P/S penicillin-streptomycin

Supplementary Table S4. Primer sequences used in this study

HGNC Symbol	HGNC official full name	NCBI Gene ID	Primer sequence (forward)	Primer sequence (reverse)	Amplicon size (bp)
ACTB	Actin beta	60	5′-ATAGCACAGCCTGGATAGCAA-3′	5′-AATCTGGCACCACACCTTCTA-3′	170
B2M	Beta-2-microglobulin	567	5′-TGGGTTTCATCCATCCGACA-3′	5′-ACGGCAGGCATACTCATCTT-3′	160
G6PD	Glucose-6-phosphate dehydrogenase	2539	5′-CAACCACATCTCCTCCCTGT-3′	5′-ATGTTGTCCCGGTTCCAGAT-3′	138
GAPDH	Glyceraldehyde-3-phosphate dehydrogenase	2597	5′-AAATGAGCCCCAGCCTTCTC-3′	5′-GTCAAGGCTGAGAACGGGAA-3′	158
GUSB	Glucuronidase beta	2990	5′-CCCACTCAGTAGCCAAGTCA-3′	5′-CACAAAACCCAGGCCAGAAA-3′	172
HMBS	Hydroxymethylbilane synthase	3145	5′-AAGACCCTAGAAACCCTGCC-3′	5′-TCCAGATGCGGGAACTTTCT-3′	92
HPRT1	Hypoxanthine phosphoribosyltransferase 1	3251	5′-ACTGAACGTCTTGCTCGAGA-3′	5′-GCTACAATGTGATGGCCTCC-3′	56
IPO8	Importin 8	10526	5′-TGAACAACAGGGAGGCTTCA-3′	5′-TTCATCCCCTTGACCCTCAC-3′	159
PGK1	Phosphoglycerate kinase 1	5230	5′-GGACAGGACCACAGAGCTAG-3′	5′-GGAGGCACATTGGAAAAGCA-3′	90
PPIA	Peptidylprolyl isomerase A	5478	5′-GGAGGCTTTGAGGTTTTGCA-3′	5′-TTAAGGTGGGCAGAGAAGGG-3′	171
TBP	TATA-box binding protein	6908	5′-AGTTCTGGGATTGTACCGCA-3′	5′-TGTGCACACCATTTTCCCAG-3′	195
TFRC	Transferrin receptor	7037	5′-AGATTCCTGGTTCGGGTGTT-3′	5′-TGAAGAGACTCACTGCTGCA-3′	113
UBC	Ubiquitin C	7316	5′-TGGGTCGCAGTTCTTGTTTG-3′	5′-ACGAAGATCTGCATTGTCAAGT-3′	60
YWHAZ	Tyrosine 3-monooxygenase/tryptophan 5-monooxygenase activation protein zeta	7534	5′-CGCCAGAACATCCAGTCATG-3′	5′-ATCATATCGCTCAGCCTGCT-3′	77

Primer sequences of the 14 housekeeping genes examined. HGNC, Human Genome Organization (HUGO) Gene Nomenclature Committee

Supplementary Table S5. Clinicopathological parameters of patients and experimental conditions for the organoids

Samples	Sex	Age	BMI	cT stage	cN stage	ypT stage	ypN stage	Histological differentiation	Tumor budding	Perineural invasion	KRAS	BRAF	MSI	Organoid RT	Viability after RT (% control)	260/280 OD ratio
Organoid 1	Male	64	24.31	T3	N2	T3	N2b	Moderately	Present	Present	Mutant	Wild	MSS	Control		1.87
Organoid 1														21 Gy	38.1	1.8
Organoid 2	Male	42	20.7	T4a	N2	NA	NA	NA	NA	NA	NA	NA	MSS	Control		1.89
Organoid 2														21 Gy	13.9	1.85
Organoid 3	Male	64	20.35	T3	N1	T3	N1a	Moderately	Absent	Absent	NA	NA	MSS	Control		1.86
Organoid 3														21 Gy	49.9	1.83

The 260/280 OD ratio was used to assess the purity of DNA and RNA. RT, radiotherapy; OD, optical density; MSS, microsatellite stable; NA, Not available.

Supplementary Table S6. Clinicopathological parameters of patients and quality of RNA extracted for the fresh frozen tissues

Samples	Condition	Sex	Age	BMI	cT stage	cN stage	ypT stage	ypN stage	Histological differentiation	Tumor budding	Perineural invasion	KRAS	BRAF	OP	CTx	Regimen	RT	Dose	TRG	MSI	DV200
Frozen 1	Pre-RT	M	66	23.47	T4b	N2b	T3	N0	Moderately	Absent	Absent	NA	NA		Yes	5-FU	Yes	50.4 Gy		NA	93.41
Frozen 1	Post-RT											Mutant	Wild	LAR	Yes	FOLFOX-4			2	MSS	90.3
Frozen 2	Pre-RT	M	67	23.79	T2	N1	T2	N0	Moderately	Present	Absent	NA	NA		Yes	Xeloda	Yes	50.4 Gy		MSS	96.99
Frozen 2	Post-RT											Wild	Wild	LAR	Yes	FOLFOX-4			3	MSS	92.54
Frozen 3	Pre-RT	M	55	20.89	T3	N2b	T3	N0	Moderately	Absent	Present	NA	NA		Yes	Xeloda	Yes	50.4 Gy		MSS	86.45
Frozen 3	Post-RT											Mutant	Wild	LAR	Yes	FOLFOX-6			3	MSS	88.34
Frozen 4	Pre-RT	M	63	15.31	T4a	N2b	T3	N0	Moderately	Absent	Present	NA	NA		Yes	5-FU	Yes	50.4 Gy		MSS	97.93
Frozen 4	Post-RT											Mutant	Wild	LAR	Yes	FOLFOX-4			3	MSS	94.47
Frozen 5	Pre-RT	M	64	23.53	T4a	N1b	T3	N0	Moderately	Absent	Absent	NA	NA		Yes	Xeloda	Yes	50.4 Gy		MSS	95.07
Frozen 5	Post-RT											Wild	Wild	LAR	Yes	FOLFOX-4			3	MSS	90.1
Frozen 6	Pre-RT	F	54	19.56	T4	N2	T3	N2a	Moderately	Absent	Present	NA	NA		Yes	5-FU	Yes	50.4 Gy		MSS	77.94
Frozen 6	Post-RT											Wild	Wild	LAR	Yes	FOLFOX-4			3	MSS	84.86
Frozen 7	Pre-RT	M	71	19.33	T3	N1a	T4a	N0	Moderately	Absent	Absent	Wild	NA		Yes	5-FU	Yes	50.4 Gy		NA	84.1
Frozen 7	Post-RT											Wild	Wild	LAR	No				4	MSS	81.54
Frozen 8	Pre-RT	M	71	16.86	T4a	N2b	T4a	N1b	Poorly	Present	Present	NA	NA		Yes	5-FU	Yes	54 Gy		MSS	96.92
Frozen 8	Post-RT											Wild	Wild	LAR	Yes	FOLFOX-4			4	MSS	92.07

DV200 is the percentage of RNA fragments > 200 nts. BMI, body mass index; OP, operative procedure; CTx, chemotherapy; RT, radiotherapy; TRG, tumor regression grade; MSI, microsatellite instability; NA, not available; LAR, Low anterior resection; 5-FU, 5-Fluorouracil; FOLFOX, Folinic acid, fluorouracil and oxaliplatin; MSS, microsatellite stable.

Supplementary Table S7. Clinicopathological parameters of patients and quality of RNA extracted for the formalin-fixed, paraffin-embedded samples

Samples	Condition	Sex	Age	BMI	cT stage	cN stage	ypT stage	ypN stage	Histological differentiation	Tumor budding	Perineural invasion	KRAS	BRAF	OP	CTx	Regimen	RT	Dose	TRG	MSI	DV200
FFPE 1	Pre-RT	F	78	19.1	T3	NA	T2	N0	Moderately	Present	Absent	NA	NA	LAR	No		No			NA	79.93
FFPE 1	Post-RT											Mutant	Wild		No		No		NA	MSI-H	69.97
FFPE 2	Pre-RT	M	71	19.63	T4b	N1b	T3	N1b	Moderately	Absent	Present	NA	NA	LAR	Yes	5-FU	Yes	50.4 Gy		NA	51.41
FFPE 2	Post-RT											Mutant	Wild		Yes	FOLFOX-4	No		4	MSS	59.18
FFPE 3	Pre-RT	F	81	23.23	T3	N2b	T3	N2a	Moderately	Absent	Present	NA	NA	AR	No		Yes	50.4 Gy		MSS	59.35
FFPE 3	Post-RT											Wild	Wild		No		No		5	MSS	38.87
FFPE 4	Pre-RT	M	79	20.7	T4	N2	T4a	N2a	Moderately	Absent	Present	NA	NA	LAR	Yes	5-FU	Yes	50.4 Gy		NA	61.49
FFPE 4	Post-RT											Wild	Wild		Yes	5-FU	No		3	MSS	67.82

DV200 is the percentage of RNA fragments > 200 nts. BMI, body mass index; OP, operative procedure; CTx, chemotherapy; RT, radiotherapy; TRG, tumor regression grade; MSI, microsatellite instability; NA, not available; LAR, Low anterior resection; MSI-H, microsatellite instability-high; 5-FU, 5-Fluorouracil; FOLFOX, Folinic acid, fluorouracil and oxaliplatin; MSS, microsatellite stable; AR, Abdominaoperineal resection.

Supplementary Table S8. TBP-1 isoform primer sequences

HGNC symbol	HGNC official name	NCBI gene ID	Primer sequence (forward)	Primer sequence (reverse)	Amplicon size (bp)
TBP-1	TATA-box binding protein	6908	5′-CACTGTTTCTTGGCGTGTGA-3′	5′-AGCATCTCCAGCACACTCTT-3′	72

HGNC, Human Genome Organization (HUGO) Gene Nomenclature Committee
